# On the Evolution of Regularized Dirac-Harmonic Maps from Closed Surfaces

**DOI:** 10.1007/s00025-020-1178-5

**Published:** 2020-03-18

**Authors:** Volker Branding

**Affiliations:** Faculty of Mathematics, University of Vienna, Oskar-Morgenstern-Platz 1, 1090 Vienna, Austria

**Keywords:** Regularized Dirac-harmonic maps, gradient flow, weak solution, 53C27, 53C43, 58E20, 58J35

## Abstract

We study the evolution equations for a regularized version of Dirac-harmonic maps from closed Riemannian surfaces. We establish the existence of a global weak solution for the regularized problem, which is smooth away from finitely many singularities. Moreover, we discuss the convergence of the evolution equations and address the question if we can remove the regularization in the end.

## Introduction and Results

Harmonic maps from Riemannian surfaces to Riemannian manifolds are a variational problem with rich structure. Due to their conformal invariance the latter share a lot of special properties. Among these are for example their regularity and the removal of isolated singularities. The existence of harmonic maps from surfaces has been established by several methods. The approach by Sacks and Uhlenbeck [32] uses a perturbation of the energy functional such that it satisfies the Palais–Smale condition. The heat flow method was successfully applied in this case by Struwe [33].

An extension of harmonic maps motivated from supersymmetric field theories in physics are *Dirac-harmonic maps* introduced in [17]. These also arise as critical points of an action functional and couple the equation for harmonic maps with spinor fields. A Dirac-harmonic map is given by a pair $(\phi ,\psi )$ consisting of a map $\phi$ and a spinor $\psi$ along that map. Moreover, Dirac-harmonic maps still belong to the class of conformally invariant variational problems. For the physics background see [21].

Taking also into account an additional curvature term in the energy functional one is led to *Dirac-harmonic maps with curvature term*, see [6, 8, 15]. Dirac-harmonic maps coupled to a two-form potential, called *Magnetic Dirac-harmonic maps*, are studied in [5] and Dirac-harmonic maps to manifolds with torsion are examined in [7].

At present, many analytical results for Dirac-harmonic maps have already been obtained. These include the regularity of solutions [16, 38, 42], a removable singularity theorem [17] and the energy identity [41]. Dirac-harmonic maps between closed surfaces are classified in [40]. Several vanishing results for Dirac-harmonic maps and their variants can be found in [10, 11, 15].

Although many analytical aspects of Dirac-harmonic maps are well understood by now, the existence question is still not answered in general. Some explicit solutions of the Euler–Lagrange equations for Dirac-harmonic maps are given in [27]. Using the Atiyah-Singer index theorem uncoupled solutions to the Euler Lagrange equations have been constructed in [1]. Namely, for a given map $\phi _0$ a spinor $\psi$ is constructed such that the pair $(\phi _0,\psi )$ is a Dirac-harmonic map. The boundary value problem for Dirac-harmonic maps was treated in [19, 20]. For a recent survey on mathematical results regarding Dirac-harmonic maps and their variants see [25].

Since Dirac-harmonic maps interpolate between harmonic maps and harmonic spinors, the existence question for Dirac-harmonic maps can be attacked from two different perspectives. On the one hand, one may use methods from spin geometry, as in [1], or one may apply methods from the analysis of harmonic maps. This of course includes the heat-flow method. However, we cannot apply it directly since the energy functional for Dirac-harmonic maps is unbounded from below.

Hence, our approach is to solve an easier problem first and to hope that one can take a suitable limit in the end. More precisely, we consider the following regularized energy functional1.1The first term is the Dirichlet energy of the map $\phi$, $\psi$ is a vector spinor and  the twisted Dirac operator acting on $\psi$. The last term is the $L^2$-norm of the covariant derivative of $\psi$. Moreover, $\varepsilon >0$ is a bookkeeping parameter. We study the $L^2$-gradient flow of $E_{\varepsilon }(\phi ,\psi )$, which is given by1.21.3with initial data $(\phi _0,\psi _0)$. Here, $\tau (\phi )$ is the tension field of the map $\phi$ and $\tilde{\Delta }$ denotes the connection Laplacian for vector spinors. Since $\psi$ is a section in the vector bundle $\Sigma M\otimes \phi ^{-1}TN$ we have to use the covariant derivative on this bundle to calculate the derivative of $\psi$ with respect to $t$, which is denoted by $\frac{\tilde{\nabla }\psi _t}{\partial t}$. The curvature terms ${\mathcal {R}}(\phi _t,\psi _t)$ and ${\mathcal {R}}_c(\phi _t,\psi _t)$ are of lower order.

Before we state our main result let us make the following observation:

### Remark 1.1

The functional $E_\varepsilon (\phi ,\psi )$ satisfies

$$\begin{aligned} -\frac{1}{4\varepsilon }\int _M|\psi |^2dM\le E_\varepsilon (\phi ,\psi )\le \infty . \end{aligned}$$

Thus, the $L^2$-norm of $\psi$ will play an important role in the study of the $L^2$-gradient flow of the functional $E_\varepsilon (\phi ,\psi )$.

Our aim is to prove a result similar to Struwe’s result [33], see also [34], for the harmonic map heat flow from surfaces. Due to the coupling between the fields $\phi$ and $\psi$ new analytical difficulties arise. Nevertheless, we will prove

### Theorem 1.2

Let *M* be a closed Riemannian surface with fixed spin structure and $N$ a compact Riemannian manifold without boundary. Suppose that

1.4 $$\begin{aligned} \int _M|\psi _t|^2dM\le c_1\int _M|\tilde{\nabla }\psi _t|^2dM \end{aligned}$$

holds for all $t\in [0,\infty )$, where $c_1>0$.

Then for any smooth initial data $(\phi _0,\psi _0)$ and $\varepsilon >0$ sufficiently large, there exists a global weak solution

$$\begin{aligned} \phi :M\times [0,\infty )\rightarrow N,\qquad \psi :M\times [0,\infty )\rightarrow \Sigma M\otimes \phi ^{-1}TN \end{aligned}$$

of (1.2) and (1.3) on $M\times [0,\infty )$, which is smooth away from at most finitely many singular points $(x_k,t_k), 1\le k\le K$ with $K=K(\varepsilon ,\phi _0,\psi _0)$. The weak solution constructed here is unique and the energy functional (1.1) of the weak solution is decreasing with respect to time.

There exists a sequence $t_k\rightarrow \infty$ such that $(\phi (\cdot ,t_k),\psi (\cdot ,t_k))$ converges weakly in $H^{1}(M,N)\times H^{1}(M,\Sigma M\otimes \phi _t^{-1}TN)$ to a regularized Dirac-harmonic map $(\phi _\infty ,\psi _\infty )$ as $k\rightarrow \infty$ suitably and strongly away from finitely many points $(x_k,t_k=\infty )$. The pair $(\phi _\infty ,\psi _\infty )$ is smooth on $M{\setminus }\{x_1,\ldots ,x_K\}$.

### Remark 1.3

1. It seems that we have to impose the condition (1.4) in order to be able to prove Theorem 1.2.
2. Unfortunately, taking the limit $\varepsilon \rightarrow 0$ after $t\rightarrow \infty$ to obtain a Dirac-harmonic map does not seem to be possible. We will see later, that both the number of singularities and the regularity of $(\phi _\infty ,\psi _\infty )$ crucially depend on $\varepsilon$.

A similar approach in the one-dimensional case was performed in [9], see also [24]. Recently, a new heat-flow approach for Dirac-harmonic maps has been studied in which the Dirac equation is considered as a constraint while the map is deformed by a heat-type equation. Several existence results using this approach could be obtained in the case of a one-dimensional domain [18] and for the domain being a compact surface with boundary [26]. The short time existence for this flow in the case of a closed manifold was recently established in [39].

The results presented in this article are part of the author’s PhD thesis [4].

We would also like to point out that several existence results for Dirac-wave maps could be established [12, 13, 23] which are Dirac-harmonic maps from a domain with a Lorentzian metric.

This article is organized as follows. After introducing the framework for Dirac-harmonic maps, we present a regularized version of Dirac-harmonic maps. Afterwards, we study the $L^2$-gradient flow of the regularized functional in Sect. 2. In Sect. 3 we establish the existence of a long-time solution and Sect. 4 then discusses the convergence of the evolution equations. In the last section we analyze the limit $\varepsilon \rightarrow 0$.

Let us now describe the setup in more detail. We suppose that *M* is a closed Riemannian spin surface and *N* a compact Riemannian manifold. Every orientable Riemannian surface admits a spin structure, the number of different spin structures can be counted by the genus of the surface. For more details on spin geometry, see the book [29]. Coordinates on *M* will be denoted by *x*, whereas coordinates on *N* will be denoted by *y*. Indices on *M* are labeled by Greek letters, whereas indices on *N* are labeled by Latin letters. We use the Einstein summation convention, which means that we will sum over repeated indices.

For a given map $\phi :M\rightarrow N$, we consider the pull-back bundle $\phi ^{-1}TN$ of *TN* and twist it with the spinor bundle $\Sigma M$. On this twisted bundle $\Sigma M\otimes \phi ^{-1}TN$ there is a metric induced from the metrics on $\Sigma M$ and $\phi ^{-1}TN$. The induced connection on $\Sigma M\otimes \phi ^{-1}TN$ will be denoted by $\tilde{\nabla }$. We will always assume that all connections are metric and free of torsion. Locally, sections of $\Sigma M\otimes \phi ^{-1}TN$, called *vector spinors*, can be expressed as

$$\begin{aligned} \psi (x)=\psi ^i(x)\otimes \frac{\partial }{\partial y^i}(\phi (x)). \end{aligned}$$

On the spinor bundle $\Sigma M$ we have the Clifford multiplication of spinors with tangent vectors, which is skew-symmetric, namely

$$\begin{aligned} \langle \psi ,X\cdot \chi \rangle _{\Sigma M}=-\langle X\cdot \psi ,\chi \rangle _{\Sigma M} \end{aligned}$$

for $\psi ,\chi \in \Gamma (\Sigma M)$ and $X\in TM$. We denote the Dirac operator on $\Sigma M$ by  and the Dirac operator on the twisted bundle by , which is given bywhere $e_\alpha$ is a local basis of $TM$. In terms of local coordinates  can be expressed aswhere $\Gamma ^i_{jk}$ are the Christoffel symbols on $N$. Since the connection on $\phi ^{-1}TN$ is metric the operator  is self-adjoint with respect to the $L^2$ norm.

We may now state the energy functional for Dirac-harmonic maps1.5which has the critical points (see [17], p. 413, Prop. 2.1):

### Proposition 1.4

The Euler–Lagrange equations for the functional $E(\phi ,\psi )$ are given by1.61.7where $\tau (\phi )$ is the tension field of the map $\phi$ and the right hand side ${\mathcal {R}}(\phi ,\psi )$ is explicitly given by

1.8 $$\begin{aligned} {\mathcal {R}}(\phi ,\psi )=\frac{1}{2}R^N(\psi ,e_\alpha \cdot \psi )d\phi (e_\alpha ) \end{aligned}$$

with $R^N$ being the Riemann curvature tensor on $N$.

In terms of local coordinates, the Euler–Lagrange equations acquire the formwhere $$R^m_{~lij}$$ are the components of the curvature tensor on $$N$$. Solutions of the system (1.6), (1.7) are called $$Dirac-harmonic maps $$ from $$M\rightarrow N$$.

In the analysis of the energy functional $E(\phi ,\psi )$ one faces the problem that it is unbounded from below, since the operator  is unbounded. To overcome these analytical difficulties, we “improve” the energy functional $E(\phi ,\psi )$ by adding a regularizing term, see (1.1). Note that we formally have

$$\begin{aligned} \lim _{\varepsilon \rightarrow 0}E_\varepsilon (\phi ,\psi )=E(\phi ,\psi ). \end{aligned}$$

Of course, we would like to keep the parameter $\varepsilon$ as small as possible. Unfortunately, in order to derive energy estimates, we have to drop this assumption.

As a next step we present the Euler–Lagrange equations for $E_\varepsilon (\phi ,\psi )$.

### Proposition 1.5

The critical points of the functional $E_\varepsilon (\phi ,\psi )$ are given by1.91.10with the curvature term

$$\begin{aligned} {\mathcal {R}}_c(\phi ,\psi )= R^N(\tilde{\nabla }_{e_\alpha }\psi ,\psi )d\phi (e_\alpha ) \in \Gamma (\phi ^{-1}TN) \end{aligned}$$

and ${\mathcal {R}}(\phi ,\psi )$ given by (1.8). Moreover, $\tilde{\Delta }$ denotes the connection Laplacian on the bundle $\Sigma M\otimes \phi ^{-1}TN$.

### Proof

For a proof, see [4], Section 2.2. $\square$

Written in local coordinates, the new terms arising from the variation of $E_\varepsilon (\phi ,\psi )$ acquire the following form:

$$\begin{aligned} {\mathcal {R}}_c(\phi ,\psi )&= R^m_{~lij}\frac{\partial }{\partial y^m}\left( \frac{\partial \phi ^l}{\partial x_\alpha }\langle \nabla _{e_\alpha }^{\Sigma M}\psi ^i,\psi ^j\rangle _{\Sigma M} +\Gamma ^j_{rs}\frac{\partial \phi ^l}{\partial x_\alpha }\langle \psi ^i,\psi ^r\rangle _{\Sigma M}\frac{\partial \phi ^s}{\partial x_\alpha }\right) ,\\ \tilde{\Delta }\psi =&\left( \Delta ^{\Sigma M}\psi ^m+2\nabla ^{\Sigma M}_{e_\alpha }\psi ^i\Gamma ^m_{ij}\frac{\partial \phi ^j}{\partial x_\alpha } +\psi ^i\Gamma ^m_{ij,p}\frac{\partial \phi ^p}{\partial x_\alpha }\frac{\partial \phi ^j}{\partial x_\alpha } +\psi ^i\Gamma ^m_{ij}\frac{\partial ^2\phi ^j}{\partial x_\alpha ^2} \right. \\&\left. +\,\psi ^i\Gamma _{ij}^k\Gamma ^m_{ks}\frac{\partial \phi ^j}{\partial x_\alpha }\frac{\partial \phi ^s}{\partial x_\alpha } \right) \otimes \frac{\partial }{\partial y^m}. \end{aligned}$$

Solutions of the system (1.9), (1.10) will be called *regularized Dirac-harmonic maps* from $M\rightarrow N$.

### Remark 1.6

On a compact Riemann surface the following terms are invariant under conformal transformations:A proof can for example be found in [17], p. 416, Lemma 3.1. In particular, this means that the functional $E(\phi ,\psi )$ is conformally invariant in dimension two. We will see later that the $L^4$-norm of $\psi$ plays an important role in the context of a removable singularity theorem. On the other hand, we note that through the regularization the conformal invariance is broken.

## Evolution Equations and Energy Estimates

We now turn to the $L^2$-gradient flow of the regularized functional $E_\varepsilon (\phi ,\psi )$:2.12.2with initial data $(\phi _0,\psi _0)$.

As $\varepsilon >0$ the system (2.1), (2.2) is clearly parabolic and the existence of a smooth short-time solution up to a time $T_\text {max}$ can be obtained by standard methods, see Theorem 3.24 in [4].

Before turning to the derivation of energy estimates let us make the following remarks.

### Lemma 2.1

There does not exist a Dirac-harmonic map from $T^2\rightarrow S^2$ with $\deg \phi =\pm 1$.

### Proof

The proof is by contradiction. Assume that $(\phi ,\psi )$ is a Dirac-harmonic map from $T^2\rightarrow S^2$ with $\deg (\phi )=\pm 1$. By the classification theorem for Dirac-harmonic maps between surfaces obtained in [40] the map $\phi$ has to be harmonic in this case. On the other hand, Eells and Wood proved in [22] that there does not exist a harmonic map from $T^2\rightarrow S^2$ of degree $\pm 1$ independently of the metrics chosen on the surfaces *M* and *N*.$\square$

### Remark 2.2

Since the degree of a map is homotopy-invariant, we cannot find a Dirac-harmonic map from $T^2\rightarrow S^2$ in the homotopy class of $\phi$ with $\deg \phi =\pm 1$. This example motivates the occurrence of singularities in the heat flow for (regularized) Dirac-harmonic maps.

### Remark 2.3

We cannot hope to find a global smooth solution of (2.1) and (2.2), as already the harmonic map heat flow develops singularities in finite time [14]. In addition, we cannot expect to find a unique solution in general since in [3, 37], solutions that are different from Struwe’s solution [33], were constructed.

In the following we will often need the following combination of quantitiesMoreover, for the further analysis it turns out to be useful to introduce the following function space with $Q=M\times [0,T)$ and $dQ=dMdt$:

$$\begin{aligned} V:=\left\{ \sup _{0\le t\le T}F(\phi _t,\psi _t)+\int _Q\left( |\nabla ^2\phi |^2+|\tilde{\nabla }^2\psi |^2 +\left| \frac{\partial \phi _t}{\partial t}\right| ^2+\left| \frac{\tilde{\nabla }\psi _t}{\partial t}\right| ^2\right) dQ<\infty \right\} \end{aligned}$$

Let $\Omega \in \mathbb {R}^2$ be a bounded domain. Then Ladyzhenskaya’s inequality holds, that is

### Lemma 2.4

Assume that $v\in H_0^{1}(\Omega )$. Then the following inequality holds:

2.3 $$\begin{aligned} \Vert v\Vert ^4_{L^4(\Omega )}\le C\Vert v\Vert ^2_{L^2(\Omega )}\Vert \nabla v\Vert ^2_{L^2(\Omega )} \end{aligned}$$

In addition, we need a local version of Ladyzhenskaya’s inequality from above. By $B_R(x)$ we denote the geodesic ball of radius $R$ around $x\in M$ and $i_M$ denotes the injectivity radius of $M$. In terms of these quantities we can formulate the following:

### Lemma 2.5

Assume that $v\in V$. Then there exists a constant *C* such that for any $R\in (0,i_M)$ the following inequality holds:

2.4 $$\begin{aligned} \int _M|\nabla v|^4 dM\le C\sup _{x\in M}\int _{B_{R}(x)}|\nabla v|^2dM\left( \int _M|\nabla ^2v|^2dM+\frac{1}{R^2}\int _M|\nabla v|^2dM\right) . \end{aligned}$$

### Proof

A proof can for example be found in [35], p. 225, Lemma 6.7. $\square$

As a first step, we want to obtain a pointwise bound for the norm of the spinor $\psi _t$. Using (2.2) we calculate2.5

### Remark 2.6

If we apply the maximum principle to (2.5), we obtain the estimate

$$\begin{aligned} |\psi _t|^2\le |\psi _0|^2e^{\frac{t}{\varepsilon }}. \end{aligned}$$

In particular, if the initial spinor $\psi _0$ vanishes, then our system (2.1) and (2.2) reduces to the harmonic map heat flow studied by Struwe in [33]. Moreover, if $\psi _t=0$ for some time $t$ then $\psi _t=0$ for all $T\ge t$.

### Lemma 2.7

Let $\psi _t\in C^2(M\times [0,T),\Sigma M\otimes \phi _t^{-1}TN)$ be a solution of (2.2) and assume that (1.4) holds. For $\varepsilon$ large enough we get a uniform bound on $\psi _t$

2.6 $$\begin{aligned} |\psi _t|^2_{L^\infty (M\times [0,T))}\le Ce^\frac{1}{\varepsilon }. \end{aligned}$$

The constant $C$ depends on $M,N,c_1$ and the $L^2$-norm of $\psi _0$.

### Proof

We already know that $\psi _t$ solves the pointwise equation (2.5). If we can also bound the $L^2$-norm of the spinor $\psi _t$ we get a uniform pointwise bound by Lemma A.1 (see the “Appendix” for its precise formulation). Thus, we calculatewhere we applied (1.4) in the last step. Hence for $\varepsilon >0$ big enough the right hand side of the above equation will be negative which gives the desired bound on the $L^2$-norm of $\psi _t$. $\square$

In the following $C$ denotes a universal constant that may change from line to line. Since our evolution equations are originating from a variational problem, we get bounds in terms of the initial data $(\phi _0,\psi _0)$.

### Lemma 2.8

Let $(\phi _t,\psi _t)\in V$ be a solution of (2.1) and (2.2). If in addition $\int _M|\psi _t|^2dM\le C$, then we have for all $t\in [0,T)$

$$\begin{aligned} \int _M(|d\phi _t|^2+\varepsilon |\tilde{\nabla }\psi _t|^2)dM+ \int _Q \big (\big |\frac{\partial \phi _t}{\partial t}\big |^2+\big |\frac{\tilde{\nabla }\psi _t}{\partial t}\big |^2\big )dMdt\le C. \end{aligned}$$

The constant *C* depends on $M,\varepsilon ,E_\varepsilon (\phi _0,\psi _0)$ and $\psi _0$.

### Proof

The inequality follows from the fact that the system (2.1), (2.2) is the $L^2$-gradient flow of the functional $E_\varepsilon (\phi ,\psi )$ and$\square$

The next Lemma is the analogue of Lemma 3.6 from [33]. We want to get local bounds of the $L^2$-norms of $d\phi _t$ and $\tilde{\nabla }\psi _t$.

### Lemma 2.9

Let $(\phi _t,\psi _t)\in V$ be a solution of (2.1) and (2.2). For $R\in (0,i_M)$ and any $(x,t)\in Q$ there holds the estimate

2.7 $$\begin{aligned} E_{\varepsilon }(\phi _t,\psi _t,B_R)\le \frac{C}{R^2} \int _Q(|d\phi _t|^2+|\psi _t|^2+\varepsilon ^2|\tilde{\nabla }\psi _t|^2)dQ +E_{\varepsilon }(\phi _0,\psi _0,B_{2R}), \end{aligned}$$

where the constant *C* only depends on *M*.

### Proof

First of all, we choose a smooth cut-off function $\eta$ with the following properties

$$\begin{aligned}&\eta \in C^\infty (M),\qquad \eta \ge 0,\qquad \eta =1~\text {on}~B_R(x_0),\\&\eta =0~\text {on}~M{\setminus } B_{2R}(x_0),\qquad |\nabla \eta |_{L^\infty }\le \frac{C}{R}, \end{aligned}$$

where again $B_R(x_0)$ denotes the geodesic ball of radius $R$ around $x_0\in M$. In addition, we choose an orthonormal basis $\{e_\alpha ,\alpha =1,2\}$ on $M$ such that $\nabla _{e_\alpha }e_\beta =\nabla _{\partial _t}e_\alpha =0$ at the considered point. By a direct calculation we findMultiplying each of the terms with the cut-off function $\eta ^2$, adding up the three terms and using the evolution Eqs. (2.1) and (2.2), we findUsing integration by parts we derive

$$\begin{aligned} \int _M\eta ^2\partial _{e_\alpha }\left\langle \frac{\partial \phi _t}{\partial t}, d\phi _t(e_\alpha )\right\rangle dM \le&C\int _M|\eta ||\nabla \eta ||\frac{\partial \phi _t}{\partial t}| |d\phi _t|dM, \\ \int _M\eta ^2\partial _{e_\alpha }\left\langle \frac{\tilde{\nabla }\psi _t}{\partial t},e_\alpha \cdot \psi _t\right\rangle dM \le&C\int _M|\eta ||\nabla \eta ||\frac{\tilde{\nabla }\psi _t}{\partial t}||\psi _t|dM,\\ \int _M\eta ^2\partial _{e_\alpha }\left\langle \frac{\tilde{\nabla } \psi _t}{\partial t},\tilde{\nabla }_{e_\alpha }\psi _t\right\rangle dM \le&C\int _M|\eta ||\nabla \eta ||\frac{\tilde{\nabla }\psi _t}{\partial t}||\tilde{\nabla }\psi _t|dM. \end{aligned}$$

Applying Young’s inequality and by the properties of the cut-off function $\eta$, we find

$$\begin{aligned} \frac{\partial }{\partial t} E_{\varepsilon }(\phi _t,\psi _t,B_R)\le \frac{C}{R^2}\int _M(|d\phi _t|^2+|\psi _t|^2+\varepsilon ^2|\tilde{\nabla }\psi _t|^2)dM. \end{aligned}$$

Integration with respect to *t* yields the result.$\square$

We can use the previous Lemma to formulate monotonicity formulas for $F(\phi _t,\psi _t,B_R)$. By Young’s inequality and the “monotonicity formula” for the local energy $E_\varepsilon (\phi _t,\psi _t,B_R)$, we get

2.8 $$\begin{aligned} F(\phi _t,\psi _t,B_R)\le 2E_\varepsilon (\phi _0,\psi _0,B_{2R})+C\frac{T}{R^2} +\frac{1}{2\varepsilon }\int _{B_R}|\psi _t|^2 dM. \end{aligned}$$

Roughly speaking, we want to make the left hand side of this inequality as small as we have to. This can be achieved by choosing the initial data $(\phi _0,\psi _0)$, the radius $R$ of the ball $B_R$ and the time $T$ appropriately. More precisely, we get the following

### Corollary 2.10

Let $(\phi _t,\psi _t)\in V$ be a solution of (2.1) and (2.2). For a positive constant $\delta _1$ there exist $R\in (0,i_M)$ and $T_1>0$ with $|\psi _t|_{L^\infty (M\times [0,T_1))}\le C$ such that

2.9 $$\begin{aligned} \sup _{\genfrac{}{}{0.0pt}{}{x\in M}{0\le t\le T_1}} F(\phi _t,\psi _t,B_R)<\delta _1. \end{aligned}$$

### Proof

From Lemma 2.9 and the bound on the norm of $\psi _t$, it follows that for any $\delta _1$ and $(\phi _0,\psi _0)$ suitably, there exists a number $R>0$ for which

2.10 $$\begin{aligned} \sup _{x\in M} \left( 2E_\varepsilon (\phi _0,\psi _0,B_{2R})+\frac{1}{2\varepsilon } \int _{B_R}|\psi _t|^2dM\right) <\frac{\delta _1}{2}. \end{aligned}$$

For $T_1=\frac{\delta _1R^2}{2C}$ we then get

2.11 $$\begin{aligned} \sup _{\genfrac{}{}{0.0pt}{}{x\in M}{0\le t\le T_1}} F(\phi _t, \psi _t,B_R(x))<\delta _1, \end{aligned}$$

such that the desired estimate holds.$\square$

In order to turn the Laplace type terms into full second derivatives, we will make use of the following Bochner type formulas:

### Lemma 2.11

(Bochner type formulas) For a map $\phi :M\rightarrow N$ and a vector spinor $\psi \in \Gamma (\Sigma M\otimes \phi ^{-1}TN)$ the following Bochner type formulas hold:

2.12 $$\begin{aligned} \int _M|\tau (\phi )|^2dM=&\int _M(|\nabla d\phi |^2 +\langle d\phi (Ric^M(e_\beta )),d\phi (e_\beta )\rangle \nonumber \\&\quad -\langle R^N(d\phi (e_\alpha ),d\phi (e_\beta ))d\phi (e_\beta ),d\phi (e_\alpha )\rangle ) dM, \end{aligned}$$

2.13 $$\begin{aligned} \int _M|\tilde{\Delta }\psi |^2dM=&\int _M(|\tilde{\nabla }^2\psi |^2 +\langle R^{E_1}(e_\alpha ,e_\beta )\tilde{\nabla }_{e_\beta }\psi , \tilde{\nabla }_{e_\alpha }\psi \rangle _{E_1}\nonumber \\&+\langle R^{E_2}(e_\alpha ,e_\beta )\psi , \tilde{\nabla }_{e_\beta } \tilde{\nabla }_{e_\alpha }\psi \rangle _{E_2})dM \end{aligned}$$

with the vector bundles $E_1=T^*M\otimes \Sigma M\otimes \phi ^{-1}TN$ and $E_2=T^*M\otimes E_1$.

### Proof

This follows from a direct calculation. $\square$

We are now able to bound the $L^2$-norm of the second derivatives of $(\phi _t,\psi _t)$ on $M\times [0,T_1)$.

### Proposition 2.12

Let $(\phi _t,\psi _t)\in V$ be a solution of (2.1) and (2.2). Choose $R>0$ and $T_1>0$ such that (2.10) and (2.11) hold. Moreover, assume that $|\psi _t|_{L^\infty (M\times [0,T_1))}\le C$. Then we have for all $t\in [0,T_1)$

2.14 $$\begin{aligned} \int _Q\big (|\nabla d\phi _t|^2+\varepsilon ^2|\tilde{\nabla }^2\psi _t|^2\big )dMdt\le C\big (1+\frac{T_1}{R^2}\big ), \end{aligned}$$

where the constant *C* depends on $M,N,\varepsilon ,\psi _0,d\phi _0$ and $\tilde{\nabla }\psi _0$.

### Proof

Using the evolution equations (2.1) and (2.2) we computeApplying Young’s inequality and estimating the terms on the right hand side, we getAs a next step we transform the Laplace type terms into second derivatives, therefore we apply the Bochner type formulas (2.12), (2.13) and find

$$\begin{aligned} \frac{\partial }{\partial t}\frac{1}{2}\int _M(&|d\phi _t|^2+\varepsilon |\tilde{\nabla }\psi _t|^2)dM +C\int _M(|\nabla d\phi _t|^2+\varepsilon ^2|\tilde{\nabla }^2\psi _t|^2)dM \\&\le C\left( \int _M(|d\phi _t|^2+|\tilde{\nabla }\psi _t|^2)dM +\int _M(|d\phi _t|^4+\varepsilon ^2|\tilde{\nabla }\psi _t|^4)dM\right) , \end{aligned}$$

where we estimated all curvature contributions. Finally, we apply the local Sobolev inequality (2.4) to $\int _M|d\phi _t|^4dM$ and $\int _M|\tilde{\nabla }\psi _t|^4dM$, which leads to

$$\begin{aligned} \frac{\partial }{\partial t}\frac{1}{2}\int _M(&|d\phi _t|^2+\varepsilon |\tilde{\nabla }\psi _t|^2)dM +C\int _M(|\nabla d\phi _t|^2+\varepsilon ^2|\tilde{\nabla }^2\psi _t|^2)dM \\ \le&\,C\left( \int _M(|d\phi _t|^2+|\tilde{\nabla }\psi _t|^2)dM +\frac{\delta _1}{R^2}\int _M(|d\phi _t|^2+\varepsilon |\tilde{\nabla }\psi _t|^2)dM\right. \\&\left. +\,\delta _1\int _M(|\nabla d\phi _t|^2+\varepsilon ^2|\tilde{\nabla }^2\psi _t|^2)dM\right) . \end{aligned}$$

Choosing $\delta _1$ small enough, the terms containing the second derivatives on the right hand side can be absorbed into the left hand side. Integrating with respect to *t* yields the result. $\square$

Using the bounds on the second derivatives, we can apply the Sobolev embedding theorem to bound $\int _Q|d\phi _t|^4dQ$ and $\int _Q|\tilde{\nabla }\psi _t|^4dQ$.

### Corollary 2.13

Let $(\phi _t,\psi _t)\in V$ be a solution of (2.1) and (2.2) with $|\psi _t|_{L^\infty (M\times [0,T_1))}\le C$. If $\sup _{(x,t)\in M\times [0,T_1)}F(\phi _t,\psi _t,B_R(x))<\delta _1$, then we have for all $t\in [0,T_1)$

2.15 $$\begin{aligned} \int _Q|d\phi _t|^4dQ\le Cf_1(t),\qquad \int _Q|\tilde{\nabla }\psi _t|^4dQ\le Cf_2(t) \end{aligned}$$

with $f_i(t)$ satisfying $f_i(t)\rightarrow 0$ as $t\rightarrow 0$ for $i=1,2$.

### Proof

The bounds follow from the Sobolev embedding in two dimensions and the previous estimates, namely

$$\begin{aligned} \int _Q|d\phi _t|^4 dQ\le C\int _Q|d\phi _t|^2dQ\int _Q|\nabla d\phi _t|^2dQ \le Cf_1(t). \end{aligned}$$

The estimate on $\int _Q|\tilde{\nabla }\psi _t|^4dQ$ can be derived by the same method. $\square$

### Corollary 2.14

For $\delta _1$ small enough and integrating over a small time interval $|t-s|\le \delta _2$, we can achieve

2.16 $$\begin{aligned} \int _s^t\int _M|d\phi _t|^4dQ \le C,\qquad \int _s^t\int _M|\tilde{\nabla }\psi _t|^4dQ\le C \end{aligned}$$

and the right hand side can be made as small as needed.

The constant *C* depends on $M,N,R,\delta _1,\delta _2,\varepsilon ,\psi _0,d\phi _0$ and $\tilde{\nabla }\psi _0$.

So far, we have derived integral estimates on $Q=M\times [0,T_1)$ of the second derivatives. In order to turn these into estimates on $M$, we have to gain control over the derivatives with respect to $t$ of the pair $(\phi _t,\psi _t)$.

### Lemma 2.15

Let $(\phi _t,\psi _t)\in V$ be a solution of (2.1) and (2.2). Then we have2.172.18for all $t\in [0,T)$.

### Proof

This follows by a direct calculation. $\square$

### Proposition 2.16

Let $(\phi _t,\psi _t)\in V$ be a solution of (2.1) and (2.2) with $|\psi _t|_{L^\infty (M\times [0,T_1))}\le C$. If $\sup _{(x,t)\in M\times [0,T_1)}F(\phi _t,\psi _t,B_R(x))<\delta _1$ is small enough, we find for $\tau >0$

2.19 $$\begin{aligned} \sup _{2\tau \le t\le T_1}\int _M\left( \left| \frac{\partial \phi (\cdot ,t)}{\partial t}\right| ^2+\left| \frac{\tilde{\nabla }\psi (\cdot ,t)}{\partial t}\right| ^2\right) dM \le C(1+\tau ^{-1}), \end{aligned}$$

where the constant $C$ depends on $M,N,R,\delta _1,\delta _2,\varepsilon ,\tau ,\psi _0,d\phi _0$ and $\tilde{\nabla }\psi _0$.

### Proof

First of all, we choose an orthonormal basis $\{e_\alpha ,\alpha =1,2\}$ on $M$ such that $\nabla _{\partial _t}e_\alpha =0$ at a considered point. Combining both equations from Lemma 2.15 we getWe have to estimate all terms on the right hand side, starting with the $A_1$ term

$$\begin{aligned} \left\langle R^N\left( d\phi _t(e_\alpha ),\frac{\partial \phi _t}{\partial t}\right) \frac{\partial \phi _t}{\partial t}, d\phi _t(e_\alpha )\right\rangle \le C|d\phi _t|^2\left| \frac{\partial \phi _t}{\partial t}\right| ^2. \end{aligned}$$

Calculating directly using the fact that $\psi _t$ is bounded uniformly we find for the $A_2$ term

$$\begin{aligned} \left| \left\langle \frac{\nabla }{\partial t}{\mathcal {R}}(\phi _t,\psi _t),\frac{\partial \phi _t}{\partial t}\right\rangle \right|&\le C\left( \left| \frac{\partial \phi _t}{\partial t}\right| ^2|d\phi _t||\psi _t|^2+|d\phi _t|\left| \frac{\partial \phi _t}{\partial t}\right| \left| \frac{\tilde{\nabla }\psi _t}{\partial t}\right| |\psi _t| +\left| \frac{\nabla }{\partial t}d\phi _t\right| \left| \frac{\partial \phi _t}{\partial t}\right| |\psi _t|^2\right) \\&\le C\left( |d\phi _t|^2\left| \frac{\partial \phi _t}{\partial t}\right| ^2+\left| \frac{\partial \phi _t}{\partial t}\right| ^2 +\left| \frac{\tilde{\nabla }\psi _t}{\partial t}\right| ^2|d\phi _t|^2\right) +\frac{1}{8}\left| \frac{\nabla }{\partial t}d\phi _t\right| ^2. \end{aligned}$$

Performing the same manipulations with the $A_3$ term, we get

$$\begin{aligned} \left| \left\langle \frac{\nabla }{\partial t}{\mathcal {R}}_c(\phi _t,\psi _t),\frac{\partial \phi _t}{\partial t}\right\rangle \right|&\le C\left( \left| \frac{\partial \phi _t}{\partial t}\right| ^2|d\phi _t||\tilde{\nabla }\psi _t||\psi _t|+ |\tilde{\nabla }\psi _t||d\phi _t|\left| \frac{\partial \phi _t}{\partial t}\right| \left| \frac{\tilde{\nabla }\psi _t}{\partial t}\right| \right. \\&\quad \left. +\,\left| \frac{\tilde{\nabla }}{\partial t}\tilde{\nabla }\psi _t\right| |d\phi _t|\left| \frac{\partial \phi _t}{\partial t}\right| |\psi _t| +|\tilde{\nabla }\psi _t|\left| \frac{\nabla }{\partial t}d\phi _t\right| \left| \frac{\partial \phi _t}{\partial t}\right| |\psi _t|\right) \\&\le C\left( |d\phi _t|^2\left| \frac{\partial \phi _t}{\partial t}\right| ^2+|\tilde{\nabla }\psi _t|^2\left| \frac{\partial \phi _t}{\partial t}\right| ^2 +\left| \frac{\tilde{\nabla }\psi _t}{\partial t}\right| ^2|d\phi _t|^2\right) \\&\quad +\frac{1}{8}\left| \frac{\nabla }{\partial t}d\phi _t\right| ^2+\frac{\varepsilon }{8}\left| \frac{\tilde{\nabla }}{\partial t}\tilde{\nabla }\psi _t\right| ^2. \end{aligned}$$

As a next step, we want to control the terms arising from interchanging covariant spinorial derivatives, namely $A_4,A_5$ and $A_6$.

$$\begin{aligned} A_4&\le C|d\phi _t|\left| \frac{\partial \phi _t}{\partial t}\right| \left| \frac{\tilde{\nabla }\psi _t}{\partial t}\right| |\tilde{\nabla }\psi _t| \le C\left( |d\phi _t|^2\left| \frac{\partial \phi _t}{\partial t}\right| ^2+\left| \frac{\tilde{\nabla }\psi _t}{\partial t}\right| ^2|\tilde{\nabla }\psi _t|^2\right) ,\\ A_5&\le C|d\phi _t|\left| \frac{\partial \phi _t}{\partial t}\right| \left| \tilde{\nabla }\frac{\tilde{\nabla }\psi _t}{\partial t}\right| \le C|d\phi _t|^2\left| \frac{\partial \phi _t}{\partial t}\right| ^2+\frac{\varepsilon }{8}\left| \tilde{\nabla }\frac{\tilde{\nabla }\psi _t}{\partial t}\right| ^2. \end{aligned}$$

Regarding $A_6$, we use the pointwise bound on $\psi _t$, interchange covariant derivatives, estimate the curvature terms and findNote that $\nabla \frac{\partial \phi }{\partial t}=\frac{\nabla }{\partial t}d\phi$, which is due to the torsion freeness of the connection. We sum up the different contributions and find the following inequality

$$\begin{aligned} \frac{\partial }{\partial t}\frac{1}{2}&\int _M\left( \left| \frac{\partial \phi _t}{\partial t}\right| ^2 +\left| \frac{\tilde{\nabla }\psi _t}{\partial t}\right| ^2\right) dM +\frac{1}{2}\int _M\left( \left| \nabla \frac{\partial \phi _t}{\partial t}\right| ^2 +\varepsilon \left| \tilde{\nabla }\frac{\tilde{\nabla }\psi _t}{\partial t}\right| ^2\right) dM \\&\le C\left( \int _M|d\phi _t|^2\left| \frac{\partial \phi _t}{\partial t}\right| ^2dM +\int _M\left| \frac{\partial \phi _t}{\partial t}\right| ^2|\tilde{\nabla }\psi _t|^2dM\right. \\&\left. \quad +\int _M\left| \frac{\tilde{\nabla }\psi _t}{\partial t}\right| ^2|\tilde{\nabla }\psi _t|^2dM +\int _M\left( \left| \frac{\partial \phi _t}{\partial t}\right| ^2+\left| \frac{\tilde{\nabla }\psi _t}{\partial t}\right| ^2\right) dM\right) . \end{aligned}$$

We used part of the second order terms on the left hand side to absorb the second order terms from the right hand side.

Integrating with respect to $t$ over the domain $\tau \le s<t\le T$ we get

$$\begin{aligned} \int _s^tdt\frac{\partial }{\partial t}&\frac{1}{2}\int _M\left( \left| \frac{\partial \phi _t}{\partial t}\right| ^2+\left| \frac{\tilde{\nabla }\psi _t}{\partial t}\right| ^2\right) dM +\frac{1}{2}\int _s^t\int _M\left( \left| \nabla \frac{\partial \phi _t}{\partial t}\right| ^2 +\varepsilon \left| \tilde{\nabla }\frac{\tilde{\nabla }\psi _t}{\partial t}\right| ^2\right) dQ \\&\le C\left( \int _s^t\int _M|d\phi _t|^2\left| \frac{\partial \phi _t}{\partial t}\right| ^2dQ +\int _s^t\int _M\left| \frac{\partial \phi _t}{\partial t}\right| ^2|\tilde{\nabla }\psi _t|^2dQ\right. \\&\quad \left. +\int _s^t\int _M\left| \frac{\tilde{\nabla }\psi _t}{\partial t}\right| ^2|\tilde{\nabla }\psi _t|^2dQ +\int _s^t\int _M\left( \left| \frac{\partial \phi _t}{\partial t}\right| ^2+\left| \frac{\tilde{\nabla }\psi _t}{\partial t}\right| ^2\right) dQ\right) . \end{aligned}$$

The last term can be bounded in terms of the initial data and the $L^2$-norm of $\psi _t$ by Lemma 2.8. We use another type of Sobolev inequality (similar to (2.4) for $|t-s|\le 1$) to bound the mixed terms like $\int _s^t\int _M|\frac{\partial \phi _t}{\partial t}|^2|\tilde{\nabla }\psi _t|^2dQ$, more precisely

$$\begin{aligned} \int _s^t\int _M|d\phi _t|^2\left| \frac{\partial \phi _t}{\partial t}\right| ^2dQ\le&\left( \int _s^t\int _M|d\phi _t|^4dQ\right) ^\frac{1}{2} \left( \sup _{s\le \theta \le t} \int _M\left| \frac{\partial \phi }{\partial t}(\cdot ,\theta )\right| ^2 dM\right. \\&\left. \quad +\int _s^t\int _M \left| \nabla \frac{\partial \phi _t}{\partial t}\right| ^2dQ\right) \end{aligned}$$

and similarly for both of the other two terms.

Choosing $t-s<\delta _2$ sufficiently small, applying the Sobolev inequality and the estimates from Corollary 2.14, we can absorb part of the right hand side in the left and obtain

$$\begin{aligned}&\int _M\left( \left| \frac{\partial \phi _t(\cdot ,t)}{\partial t}\right| ^2+\left| \frac{\tilde{\nabla }\psi _t(\cdot ,t)}{\partial t}\right| ^2\right) dM\\&\quad \le \inf _{t-\delta _2\le s \le t}C\int _M\left( \left| \frac{\partial \phi _t(\cdot ,s)}{\partial t}\right| ^2+\left| \frac{\tilde{\nabla }\psi _t(\cdot ,s)}{\partial t}\right| ^2\right) dM+C. \end{aligned}$$

Finally, we estimate the infimum by the mean value, more precisely

$$\begin{aligned}&\sup _{2\tau \le t\le T_1}\int _M\left( \left| \frac{\partial \phi (\cdot ,t)}{\partial t}\right| ^2+\left| \frac{\tilde{\nabla }\psi (\cdot ,t)}{\partial t}\right| ^2\right) dM\\&\quad \le C(1+\tau ^{-1})\int _s^t\int _M\left( \left| \frac{\partial \phi _t}{\partial t}\right| ^2+\left| \frac{\tilde{\nabla }\psi _t}{\partial t}\right| ^2\right) dQ+C\\&\quad \le C(1+\tau ^{-1}). \end{aligned}$$

Hence, we get the desired bound. $\square$

### Corollary 2.17

Let $(\phi _t,\psi _t)\in V$ be a solution of (2.1) and (2.2). Assume $|\psi _t|_{L^\infty (M\times [0,T_1))}\le C$ and $\sup _{(x,t)\in M\times [0,T_1)}F(\phi _t,\psi _t,B_R(x))<\delta _1$. Then we have

2.20 $$\begin{aligned} \int _M(|\nabla ^2\phi (\cdot ,t)|^2+\varepsilon ^2|\tilde{\nabla }^2\psi (\cdot ,t)|^2)dM\le C, \end{aligned}$$

where the constant $C$ depends on $M,N,R,\delta _1,\delta _2,\varepsilon ,\tau ,\psi _0,d\phi _0$ and $\tilde{\nabla }\psi _0$.

### Proof

With the help of the previous estimates we can now bound the full second derivatives of $(\phi _t,\psi _t)$ in $L^2$. By the evolution equations (2.1), (2.2) and Young’s inequality, we findThe assertion then follows from applying the local Sobolev inequality (2.4) and the Bochner formulas (2.12), (2.13) with $\delta _1$ small enough. $\square$

### Corollary 2.18

(Higher regularity) Suppose the pair $(\phi _t,\psi _t)$ is a weak solution of (2.1) and (2.2). The pair $(\phi _t,\psi _t)$ is smooth as long as $\delta _1,\delta _2$ are small enough.

### Proof

Since we have a bound on the $L^2$-norm of the second derivatives of $\phi _t$ and $\psi _t$ by (2.17), we can apply the Sobolev embedding theorem and get that both $|d\phi _t|\in L^p$ and $|\tilde{\nabla }\psi _t|\in L^p$ for $p<\infty$. From the evolution equations (2.1) and (2.2) we may conclude that $\big |\frac{\partial \phi _t}{\partial t}\big |,|\nabla ^2\phi _t|\in L^p$ and also $\big |\frac{\tilde{\nabla }\psi _t}{\partial t}\big |,|\tilde{\nabla }^2\psi _t|\in L^p$. By the regularity theory for parabolic partial differential equations we obtain that $|d\phi _t|$ and $|\tilde{\nabla }\psi _t|$ are Hölder continuous, see [28], Theorem IV.9.1 and Lemma II.3.3. At this point the smoothness of the pair $(\phi ,\psi )$ follows from a standard bootstrap argument using Schauder theory, for more details see Theorem 3.24 in [4]. $\square$

## Long-Time Existence and Singularities

In this section we establish the existence of a long-time solution to the evolution equations. Thus, we first of all derive a uniqueness and stability result. To avoid the problem of identifying sections in different vector bundles, we will make use of the Nash embedding theorem. Hence, assume $N\subset \mathbb {R}^q$ isometrically and denote the isometric embedding by $\iota$. Then, $u=\iota \circ \phi :M\rightarrow \mathbb {R}^q$ can be thought of as a vector-valued function. The vector spinor $\psi$ turns into a vector of usual spinors $\psi =(\psi ^1,\ldots ,\psi ^q)$ with $\psi ^i\in \Gamma (\Sigma M),~i=1,\ldots ,q$. The condition that $\psi$ is along the map $\phi$ is encoded by

$$\begin{aligned} \sum _{i=1}^q\nu _i\psi ^i=0 \qquad \text {for a normal vector } \nu \in \mathbb {R}^q \text { at } \phi (x). \end{aligned}$$

Now, the function $u$ satisfies the following equation:

3.1 $$\begin{aligned} \big (\frac{\partial }{\partial t}-\Delta \big )u=&-\mathbb {I}_u(du,du) -P(\mathbb {I}(du(e_\alpha ),e_\alpha \cdot \psi ),\psi )-\varepsilon B(du,\psi ,du,\psi ) \nonumber \\&+\varepsilon P(\mathbb {I}(du(e_\alpha ),\psi ),\nabla _{e_\alpha }\psi ) -\varepsilon P(\mathbb {I}(du(e_\alpha ),\nabla _{e_\alpha }\psi ),\psi ) \end{aligned}$$

with the initial condition $u_0=\iota (\phi _0)$ and

$$\begin{aligned} B_u(du,\psi ,du,\psi )=&\frac{\partial u^i}{\partial x_\alpha }\frac{\partial u^k}{\partial x_\alpha }\langle \psi ^{l},\psi ^{j}\rangle \big (P(\mathbb {I}_u(\partial _{y^i},\partial _{y^m}),\partial _{y^j})\Gamma ^m_{kl}\\&-P(\mathbb {I}_u(\partial _{y^i},\partial _{y^j}),\partial _{y^m})\Gamma ^m_{kl}\big ). \end{aligned}$$

For the spinor $\psi \in \Gamma (\Sigma M\otimes T\mathbb {R}^q)$, we get the following evolution equation3.2with the initial condition $\psi _0=d\iota (\psi '_0)$, where $\psi '_0\in \Gamma (\Sigma M\otimes \phi _0^{-1}TN)$. For a derivation of (3.1) and (3.2) see [4], Section 3.4. Here, $\mathbb {I}$ is the second fundamental form of the embedding and $P$ denotes the shape operator. By projecting to a tubular neighborhood $\tilde{N}$ of $\iota (N)\subset \mathbb {R}^q$ we can think of $\mathbb {I}$ as a vector-valued function in $\mathbb {R}^q$. For more details, see [31], p. 132.

Assuming $|\psi _t|_{L^\infty (M\times [0,T))}\le C$ and $\sup _{(x,t)\in M\times [0,T)}F(\phi _t,\psi _t,B_R(x))<\delta _1$ we obtain by Corollary 2.17 and the Sobolev embedding theorem that

3.3 $$\begin{aligned} \int _M|du|^4dM \le C,\qquad \int _M|\nabla \psi |^4dM\le C \end{aligned}$$

such that we can prove the following

### Proposition 3.1

(Stability and Uniqueness) Let $(\phi ,\psi )\in V$ and $(\xi ,\chi )\in V$ be solutions of (2.1) and (2.2), where $\psi \in \Gamma (\Sigma M\otimes \phi ^{-1}TN)$ and moreover $\chi \in \Gamma (\Sigma M\otimes \xi ^{-1}TN)$. In addition, suppose that $|\psi |_{L^\infty (M\times [0,T))}\le C$ and $|\chi |_{L^\infty (M\times [0,T))}\le C$. If the initial data coincide, $(\phi _0,\psi _0)=(\xi _0,\chi _0)$, then we have $(\phi ,\psi )=(\xi ,\chi )$ throughout $M\times [0,T)$.

### Proof

We follow [35], p. 235. We regard $u,v$ as vector-valued functions in $\mathbb {R}^q$ with $u=\iota \circ \phi ,v=\iota \circ \xi$. The spinors $\psi$ and $\chi$ are defined along the maps $u$ and $v$. We set

$$\begin{aligned} h(x,t)=(h_1(x,t),h_2(x,t))=(u(x,t)-v(x,t),\psi (x,t)-\chi (x,t)). \end{aligned}$$

First, we study the evolution of $h_1$ and $h_2$ separately and add up both contributions in the end. We compute using (3.1)

$$\begin{aligned} \frac{\partial }{\partial t}\frac{1}{2}\int _M&|h_1|^2dM=-\int _M|dh_1|^2dM - \int _M\langle \mathbb {I}_u(du,du)-\mathbb {I}_v(dv,dv),h_1\rangle dM\\&-\int _M\langle h_1,P(\mathbb {I}_u(du(e_\alpha ),e_\alpha \cdot \psi ),\psi )-P(\mathbb {I}_v(dv(e_\alpha ), e_\alpha \cdot \chi ),\chi )\rangle dM\\&-\varepsilon \int _M\langle h_1,P(\mathbb {I}_u(du(e_\alpha ),\nabla _{e_\alpha }\psi ),\psi )-P(\mathbb {I}_v(dv(e_\alpha ), \nabla _{e_\alpha }\chi ),\chi )\rangle dM\\&+\varepsilon \int _M\langle h_1,P(\mathbb {I}_u(du(e_\alpha ),\psi ),\nabla _{e_\alpha }\psi )-P(\mathbb {I}_v(dv(e_\alpha ), \chi ),\nabla _{e_\alpha }\chi )\rangle dM\\&-\varepsilon \int _M\langle h_1,B_u(du,\psi ,du,\psi )-B_v(dv,\chi ,dv,\chi )\rangle dM. \end{aligned}$$

We estimate the right hand side in terms of $h_1$ and $h_2$, where we apply the pointwise bounds on $\psi$ and $\chi$. We will sketch this in detail for the term with the second fundamental forms, the other terms can then be treated similarly. Rearranging

$$\begin{aligned} \mathbb {I}_u(du,du)-\mathbb {I}_v(dv,dv)= (\mathbb {I}_u-\mathbb {I}_v)(du,du)+\mathbb {I}_v(du-dv,du)+\mathbb {I}_v(dv,du-dv) \end{aligned}$$

and applying the mean value theorem, we find

$$\begin{aligned} |\langle \mathbb {I}_u(du,du)-\mathbb {I}_v(dv,dv),u-v\rangle |\le C(|du|^2|u-v|^2+(|du|+|dv|)|h_1||dh_1|). \end{aligned}$$

Hence, we obtain the bound

$$\begin{aligned} |\langle \mathbb {I}_u(du,du)-\mathbb {I}_v(dv,dv),h_1\rangle |&\le C(|h_1|^2(|du|^2+|dv|^2)+\frac{1}{8}|dh_1|^2. \end{aligned}$$

Using the pointwise bound on the spinors we find

$$\begin{aligned} |\langle h_1,P(\mathbb {I}_u(du(e_\alpha ),e_\alpha \cdot \psi )&,\psi )-P(\mathbb {I}_v(dv(e_\alpha ), e_\alpha \cdot \chi ),\chi )\rangle |\\&\le C(|du|^2|h_1|^2+|h_1|^2+|dv|^2|h_2|^2)+\frac{1}{8}|dh_1|^2 \end{aligned}$$

and

3.4 $$\begin{aligned} |\langle h_1,&P(\mathbb {I}_u(du(e_\alpha ),\psi ),\nabla _{e_\alpha }\psi ) -P(\mathbb {I}_v(dv(e_\alpha ),\chi ),\nabla _{e_\alpha }\chi )\rangle | \nonumber \\&\le C(|h_1|^2(|du|^2+|\nabla \psi |^2+|dv|^2)+|h_2|^2|\nabla \psi |^2) +\frac{1}{8}|dh_1|^2+\frac{1}{8}|\nabla h_2|^2. \end{aligned}$$

Note that the contribution

$$\begin{aligned} \varepsilon \langle h_1,P(\mathbb {I}_u(du(e_\alpha ),\nabla _{e_\alpha }\psi ), \psi )-P(\mathbb {I}_v(dv(e_\alpha ),\nabla _{e_\alpha }\chi ),\chi )\rangle \\ \end{aligned}$$

can be estimated the same way as (3.4). In addition, we have

$$\begin{aligned} |\langle h_1,B_u(du,\psi ,du,\psi )-B_v(dv,\chi ,dv,\chi )\rangle |&\le C(|h_1|^2(|du|^2+|dv|^2)+|h_2|^2|dv|^2)\\&\quad +\frac{1}{8}|dh_1|^2. \end{aligned}$$

We now turn to the function $h_2$. With the help of (3.2) we find3.5The other terms involving the second fundamental form vanish since $\mathbb {I}\perp \psi$ and we may estimateTo estimate the last term in (3.5), we rearrange and estimate

$$\begin{aligned}&|\langle \psi -\chi ,(\nabla _{e_\alpha }\mathbb {I}_u)(du(e_\alpha ),\psi )-(\nabla _{e_\alpha } \mathbb {I}_v)(dv(e_\alpha ),\chi )\rangle |\\&\quad \le C(|h_1|^2|du|^2+|dv|^2|h_2|^2)+\frac{1}{8}|dh_1|^2. \end{aligned}$$

Adding up the inequalities for $|h_1|^2$ and $|h_2|^2$ and applying the Sobolev embedding theorem, we find

$$\begin{aligned}&\frac{\partial }{\partial t}\frac{1}{2}\int _M(|h_1|^2+|h_2|^2)dM +\frac{1}{2}\int _M(|dh_1|^2+\varepsilon |\nabla h_2|^2)dM \\&\quad \le C\int _M(|h_1|^2+|h_2|^2)dM+C\int _M(|h_1|^2+|h_2|^2) (|du|^2+|dv|^2+|\nabla \psi |^2)dM. \end{aligned}$$

Applying (3.3) and using the Sobolev embedding theorem the last term on the right hand side can be estimated as

$$\begin{aligned} C\int _M|h_1|^2|du|^2dM&\le C\left( \int _M|h_1|^4dM\right) ^\frac{1}{2} \left( \int _M|du|^4dM\right) ^\frac{1}{2} \\&\le C\left( \int _M|h_1|^2dM\right) ^\frac{1}{2}\left( \int _M|dh_1|^2dM\right) ^\frac{1}{2} \\&\le \frac{1}{8}\int _M|dh_1|^2dM+C\int _M|h_1|^2dM \end{aligned}$$

and the other contributions can be treated similarly. Hence, we find

$$\begin{aligned} \frac{\partial }{\partial t}\frac{1}{2}&\int _M(|h_1|^2+|h_2|^2)dM\le C\int _M(|h_1|^2+|h_2|^2)dM. \end{aligned}$$

Integrating with respect to $t$ and using that $h_1(0)=h_2(0)=0$ we may follow that $h_1=h_2=0$ for all $t\in [0,T)$, which proves the claim. $\square$

### Proposition 3.2

(Long-time Existence) Let $(\phi _t,\psi _t)\in V$ be a solution of (2.1) and (2.2). Assume that $|\psi _t|_{L^\infty (M\times [0,T))}\le C$. Then the evolution equations admit a unique weak solution for $0\le t<\infty$.

### Proof

The first singular time $T_0$ is characterized by the condition

$$\begin{aligned} \limsup _{t\rightarrow T_0}F(\phi _t,\psi _t,B_R(x))\ge \delta _1. \end{aligned}$$

Since we have $\partial _t\phi ,\tilde{\nabla }_t\psi \in L^2(M\times [0,T_0))$ and also $F(\phi _t,\psi _t)\le CF(\phi _0,\psi _0)+C$ for $0<t<T_0$, there exists

$$\begin{aligned} (\phi (\cdot ,T_0),\psi (\cdot ,T_0))\in H^{1}(M,N)\times H^{1}(M,\Sigma M\otimes \phi _t^{-1}TN) \end{aligned}$$

such that

$$\begin{aligned} (\phi (\cdot ,t),\psi (\cdot ,t))\rightarrow (\phi (\cdot ,T_0),\psi (\cdot ,T_0)) \end{aligned}$$

weakly in $H^{1}(M,N)\times H^{1}(M,\Sigma M\otimes \phi _t^{-1}TN)$ as *t* approaches $T_0$. In particular, we have

$$\begin{aligned} F(\phi _{T_0},\psi _{T_0})\le \liminf _{s\rightarrow t} CF(\phi _s,\psi _s) +C\le CF(\phi _t,\psi _t)+C,\qquad 0\le t\le T_0. \end{aligned}$$

Now let $(\tilde{\phi }_t,\tilde{\psi }_t):(M\times [T_0,T_0+T_1)\rightarrow N)\times (M\times [T_0,T_0+T_1)\rightarrow \Sigma M\otimes \phi _t^{-1}TN)$ be a solution of (2.1) and (2.2). Assume that $(\tilde{\phi },\tilde{\psi })(x,t)=(\phi ,\psi )(x,t)$. We define

$$\begin{aligned} (\hat{\phi }_t,\hat{\psi }_t)= {\left\{ \begin{array}{ll} (\phi _t,\psi _t), &{}\qquad 0\le t\le T_0,\\ (\tilde{\phi }_t,\tilde{\psi }_t), &{}\qquad T_0\le t\le T_0+T_1. \end{array}\right. } \end{aligned}$$

Now $(\hat{\phi }_t,\hat{\psi }_t):(M\times [0,T_0+T_1)\rightarrow N)\times (M\times [0,T_0+T_1)\rightarrow \Sigma M\times \hat{\phi }_t^{-1}TN)$ is a weak solution of (2.1) and (2.2). By iteration, we obtain a weak solution $(\phi _t,\psi _t)$ on a maximal time interval $T_0+\delta$ for some $\delta >0$. If $T_0+\delta <\infty$ then by the above argument the solution $(\phi _t,\psi _t)$ may be extended to infinity, hence $T_0+\delta =\infty$. The uniqueness follows from Proposition (3.1). $\square$

### Proposition 3.3

Assume $(\phi _t,\psi _t)$ is a solution of (2.1) and (2.2) satisfying (1.4) and $\varepsilon$ sufficiently large. There are only finitely many singular points $(x_k,t_k),1\le k\le K$. The number $K$ depends on $M,\varepsilon ,\psi _0,d\phi _0$ and $\tilde{\nabla }\psi _0$.

### Proof

We follow the presentation in [30], p. 138, for the harmonic map heat flow. We assume that $T_0>0$ is the first singular time and define the singular set as

3.6 $$\begin{aligned} S(\phi ,\psi ,T_0)=\bigcap _{R>0}\big \{ x\in M\mid \limsup _{t\rightarrow T_0}F(\phi _t,\psi _t,B_R(x))\ge \delta _1 \big \}. \end{aligned}$$

Now, let $\{x_j\}^K_{j=1}$ be any finite subset of $S(\phi ,\psi ,T_0)$. Then we have for $R>0$

$$\begin{aligned} \limsup _{t\rightarrow T_0}\int _{B_R(x_j)}(|d\phi |^2+\varepsilon |\tilde{\nabla }\psi |^2)dM\ge \delta _1,\qquad 1\le j\le K. \end{aligned}$$

By (2.8) we have the following local inequality for the quantity $F(\phi _t,\psi _t,B_R)$

3.7 $$\begin{aligned} F(\phi _t,\psi _t,B_R(x))\le 2E_\varepsilon (\phi _0,\psi _0,B_{2R}(x))+\delta _3\frac{T}{R^2} +\frac{1}{\varepsilon }\int _{B_R}|\psi _t|^2dM \end{aligned}$$

with $\delta _3=C\int _M(|d\phi _t|^2+\varepsilon ^2|\tilde{\nabla }\psi _t|^2+|\psi _t|^2)dM$. Since $E_\varepsilon (\phi _t,\psi _t)\le E_\varepsilon (\phi _0,\psi _0)$ we obtain

$$\begin{aligned}&-\frac{1}{\sqrt{2}}\left( \int _M|\psi _t|^2dM\right) ^\frac{1}{2} \left( \int _M|\tilde{\nabla }\psi _t|^2dM\right) ^\frac{1}{2} +F(\phi _t,\psi _t) \\&\quad \le F(\phi _0,\psi _0)+\frac{1}{\sqrt{2}}\left( \int _M|\psi _0|^2dM\right) ^\frac{1}{2} \left( \int _M|\tilde{\nabla }\psi _0|^2dM\right) ^\frac{1}{2}. \end{aligned}$$

Recall that by assumption (1.4) we have,

$$\begin{aligned} \int _M|\psi _t|^2dM\le \delta _5\int |\tilde{\nabla }\psi |^2dM, \end{aligned}$$

where we have renamed the positive constant $c_1$ to $\delta _5$. From this we obtain the global estimate (with $\varepsilon$ suitably large)

$$\begin{aligned} F(\phi _t,\psi _t)\le \delta _4 F(\phi _0,\psi _0) \end{aligned}$$

for a positive constant $\delta _4=\frac{\max \{\frac{\sqrt{2}}{\delta _5}+\varepsilon ,1\}}{\min \{\frac{-\sqrt{2}}{\delta _5}+\varepsilon ,1\}}$. We choose $R>0$ such that all the $B_{2R}(x_j),1\le j\le K$ are mutually disjoint and small enough to have

$$\begin{aligned} \frac{1}{\varepsilon }\int _{B_R}|\psi _t|^2dM\le \frac{\delta _1}{4}. \end{aligned}$$

Then, we have by (3.7)

$$\begin{aligned} K\delta _1&\le \sum _{j=1}^K\limsup _{t\rightarrow T_0} F(\phi _t,\psi _t,B_R(x_j)) \\&\le \sum _{j=1}^K\left( \limsup _{t\rightarrow T_0} 2E_\varepsilon (\phi _\tau , \psi _\tau ,B_{2R}(x_j))+\frac{\delta _1}{2}\right) \\&\le 2E_\varepsilon (\phi _\tau ,\psi _\tau )+\frac{K\delta _1}{2} \\&\le 2E_\varepsilon (\phi _0,\psi _0)+\frac{K\delta _1}{2} \end{aligned}$$

for any $\tau \in [T_0-\frac{\delta _1R^2}{4\delta _3},T_0]$. We conclude that

$$\begin{aligned} K\le 4\frac{E_\varepsilon (\phi _0,\psi _0)}{\delta _1}, \end{aligned}$$

which implies the finiteness of the singular set $S(\phi ,\psi ,T_0)$. Our next aim is to show that there are only finitely many singular spatial points. Therefore we set

$$\begin{aligned} \tilde{M}=M{\setminus }\bigcup _{1\le j\le K}B_{2R}(x_j) \end{aligned}$$

and in addition, we calculate

3.8 $$\begin{aligned} F(\phi _{T_0},\psi _{T_0})&=\lim _{R\rightarrow 0} F(\phi _{T_0},\psi _{T_0},\tilde{M})\nonumber \\&\le \lim _{R\rightarrow 0}\limsup _{t\rightarrow T_0} F(\phi _{t},\psi _{t},\tilde{M})\nonumber \\&=F(\phi _t,\psi _t)-\lim _{R\rightarrow 0}\sum _{j=1}^K\liminf _{t\rightarrow T_0} F(\phi _t,\psi _t,B_{2R}(x_j))\nonumber \\&\le \delta _4 F(\phi _0,\psi _0)-\lim _{R\rightarrow 0}\sum _{j=1}^K\limsup _{t\rightarrow T_0} F(\phi _t,\psi _t,B_{R}(x_j))\nonumber \\&\le \delta _4 F(\phi _0,\psi _0)-K\delta _1. \end{aligned}$$

Now suppose $T_0<\cdots <T_j$ are *j* singular times and by $K_0,\ldots ,K_j$ we denote the number of singular points at each singular time. Set

$$\begin{aligned} (\phi _i,\psi _i)=\lim _{t\rightarrow T_i}(\phi _t,\psi _t),0\le i\le j. \end{aligned}$$

By iterating (3.8) we get

$$\begin{aligned} F(\phi _j,\psi _j)&\le \delta _4 F(\phi _{j-1},\psi _{j-1})-\delta _1 K_{j-1} \\&\le \delta _4^2 F(\phi _{j-2},\psi _{j-2})-\delta _1( K_{j-1}+\delta _4 K_{j-2})\\&\le \ldots \\&\le \delta _4^jF(\phi _0,\psi _0)-\delta _1\sum _{i=0}^{j-1}K_i\delta _4^{j-i-1}, \end{aligned}$$

which can be rearranged as

3.9 $$\begin{aligned} \sum _{i=0}^{j-1}K_i\delta _4^{-i-1}\le \frac{F(\phi _0,\psi _0)}{\delta _1}. \end{aligned}$$

We conclude that there are only finitely many singularities. $\square$

### Remark 3.4

If we compare the bound on the number of singularities of the regularized Dirac-harmonic map heat flow with the bound on the number of singularities in the harmonic map heat flow, then we realize that the former can encounter more singularities. In the case of the harmonic map heat flow we would have $\delta _4=1$ and $F(\phi _0,\psi _0)=\frac{1}{2}\int _M|d\phi _0|^2$, which lowers the upper bound in (3.9).

## Convergence and Blowup Analysis

In this section we discuss the convergence of the evolution equations (2.1) and (2.2). In addition, we address the problem of blowing up the singular points.

### Proposition 4.1

Let $(\phi _t,\psi _t)\in V$ be a solution of (2.1) and (2.2). Moreover, assume that $|\psi _t|_{L^\infty (M\times [0,\infty ))}\le C$. Then the pair $(\phi _t,\psi _t)$ converges weakly in $H^{1}(M,N)\times H^{1}(M,\Sigma M\otimes \phi _t^{-1}TN)$ and strongly in the space $W^{2,2}_{loc}(M{\setminus }\{x_k,t_k=\infty \},N)\times W^{2,2}_{loc}(M{\setminus }\{x_k,t_k=\infty \},\Sigma M\otimes \phi _t^{-1}TN)$ to a regularized Dirac-harmonic map. The limiting map $(\phi _\infty ,\psi _\infty )$ is smooth on $M{\setminus }\{x_1,\ldots ,x_k\}$.

### Proof

Since we have a uniform bound on the $L^2$-norm of the $t$ derivatives of $(\phi _t,\psi _t)$ by Lemma 2.8, we can achieve for $t_m\rightarrow \infty$ suitably

$$\begin{aligned} \int _M\left( \left| \frac{\partial \phi _t}{\partial t}\right| ^2+\left| \frac{\tilde{\nabla }\psi _t}{\partial t}\right| ^2\right) dM\big |_{t=t_m}\rightarrow 0 \end{aligned}$$

and in addition, we suppose that $T=\infty$ is non-singular

$$\begin{aligned} \limsup _{t\rightarrow \infty } (\sup _{x\in M}F(\phi _t,\psi _t,B_R(x)))<\delta _1 \end{aligned}$$

for some $R>0$. By (2.17) we have a bound on the second derivatives

$$\begin{aligned} \int _M\big (|\nabla ^2\phi |^2(\cdot ,t_m)+\varepsilon ^2|\tilde{\nabla }^2\psi |^2(\cdot ,t_m)\big )dM\le C \end{aligned}$$

and due to the Rellich–Kondrachov embedding theorem we may assume that

$$\begin{aligned} \phi (\cdot ,t_m)\rightarrow \phi _\infty&\qquad \text {strongly in}~W^{1,p}(M,N), \\ \psi (\cdot ,t_m)\rightarrow \psi _\infty&\qquad \text {strongly in}~W^{1,p}(M,\Sigma M\otimes \phi _{t_m}^{-1}TN) \end{aligned}$$

for any $p<\infty$. But then by (2.1) and (2.2) we get convergence of the evolution equations4.14.2in $L^2$, the pair $(\phi _\infty ,\psi _\infty )$ is a regularized Dirac-harmonic map, which satisfies $(\phi _\infty ,\psi _\infty )\in W^{2,2}(M,N)\times W^{2,2}(M,\Sigma M\otimes \phi _\infty ^{-1}TN).$

If $T=\infty$ is singular, meaning that at the points $\{x_1,\ldots ,x_k\}$

$$\begin{aligned} \limsup _{t\rightarrow \infty } F(\phi _t,\psi _t,B_R(x_j))\ge \delta _1,\qquad 1\le j\le k \end{aligned}$$

for all $R>0$, then for suitable numbers $t_m\rightarrow \infty$ the family $(\phi _{t_m},\psi _{t_m})$ will be bounded in $W^{2,2}_{loc}(M,N)\times W^{2,2}_{loc}(M,\Sigma M\otimes \phi _{t_m}^{-1}TN)$ on the set $M{\setminus }\{x_1,\ldots ,x_k\}$. Consequently, the family $(\phi _{t_m},\psi _{t_m})$ will accumulate as follows

$$\begin{aligned}&\phi _\infty :M{\setminus }\{x_1,\ldots ,x_k\}\rightarrow N, \\&\psi _\infty :M{\setminus }\{x_1,\ldots ,x_k\}\rightarrow \Sigma (M{\setminus }\{x_1,\ldots ,x_k\})\otimes \phi _{\infty }^{-1} TN. \end{aligned}$$

We set $\tilde{M}:=M{\setminus }\{x_1,\ldots ,x_k\}$. Concerning the regularity of $(\phi _\infty ,\psi _\infty )$ on $\tilde{M}$, we have $\phi _\infty \in W^{1,p}_{loc}(\tilde{M},N)$ for any $0<p<\infty$, since $\phi _\infty \in W^{2,2}_{loc}(\tilde{M},N)$. In addition, we have $\psi _\infty \in W^{2,2}_{loc}(\tilde{M},\Sigma \tilde{M}\otimes \phi _\infty ^{-1}TN)$ and consequently also $\psi _\infty \in W^{1,p}_{loc}(\tilde{M},\Sigma \tilde{M}\otimes \phi _\infty ^{-1}TN)$ for any $0<p<\infty$. Hence, the right hand sides of both (4.1) and (4.2) are in $L^p_{loc}$ for $2<p<\infty$. Writing $\tau (\phi )=\Delta \phi +\Gamma (\phi )(d\phi ,d\phi )$ and by elliptic estimates for second order operators we then get $\phi _\infty \in W^{2,p}_{loc}(\tilde{M},N)$ for any $0<p<\infty$. The smoothness of $(\phi _\infty ,\psi _\infty )$ then follows from a standard bootstrap argument. $\square$

This completes the proof of Theorem 1.2.

Our next aim is to get a better understanding of the singular points $(x_k,t_k)$. In the case of the harmonic map heat flow one can perform a blowup analysis, which finally leads to the “bubbling off of harmonic spheres”, see for example [33]. The important ingredient in that calculation is the fact that one can perform a parabolic rescaling of the evolution equation for harmonic maps. Thus, let us analyze the scaling of the regularized Dirac-harmonic heat flow.

### Remark 4.2

By regularizing the functional $E(\phi ,\psi )$, we haven broken the conformal invariance and consequently the evolution equations for $(\phi _t,\psi _t)$ do not scale in a “nice” way. Nevertheless, it is possible to do a rescaling if one allows to rescale $\varepsilon$ as well. It is easy to see that the evolution equations (2.1) and (2.2) are invariant under the following rescaling

4.3 $$\begin{aligned} \phi (x,t)\rightarrow&\phi (x_0+Rx,t_0+R^2t), \nonumber \\ \psi (x,t)\rightarrow&\sqrt{R}\psi (x_0+Rx,t_0+Rt),\nonumber \\ \varepsilon \rightarrow&\frac{\varepsilon }{R} \end{aligned}$$

for $R>0$. A dimensional analysis of the evolution equation for $\psi$ also motivates to rescale $\varepsilon$. Note that the two evolution equations scale differently. The evolution equation for $\phi$ scales like a heat type equation, whereas the evolution equation for $\psi$ scales like a first order evolution equation. However, it seems impossible to justify the rescaling of $\varepsilon$ at a rigorous level.

### Remark 4.3

When analyzing the bubbling of Dirac-harmonic maps, it is important to have control over the energy of the bubbles, such that now concentration phenomena can happen. This control is usually given by what is called *energy identity*. For Dirac-harmonic maps the energy identity was established in [41], p. 131.

### Definition 4.4

Let $(\phi _k,\psi _k):M\rightarrow N$ be a sequence of smooth Dirac-harmonic maps with uniformly bounded energy

$$\begin{aligned} \int _M(|d\phi _k|^2+|\psi _k|^4)dM\le C \end{aligned}$$

and furthermore assume that $(\phi _k,\psi _k)$ converges weakly to a Dirac-harmonic map $(\phi ,\psi )$ in $H^{1}(M,N)\times L^4(\Sigma M\otimes T\mathbb {R}^q)$. Then we call

$$\begin{aligned} S:=\bigcap _{R>0}\{x\in M\mid \liminf _{k\rightarrow \infty }\int _{B_R(x)}(|d\phi _k|^2+|\psi _k|^4)dM>\delta \} \end{aligned}$$

the blow-up set of $\{\phi _k,\psi _k\}$.

Note that the blow-up set for Dirac-harmonic maps differs from the blow-up set for regularized Dirac-harmonic maps (3.6) that we encountered when studying the evolution equations.

## Removing the Regularization

In this section we analyze the limit $\varepsilon \rightarrow 0$. We have seen that the regularized Dirac-harmonic map heat flow converges to a smooth regularized Dirac-harmonic map $(\phi _\infty ,\psi _\infty )$ on $M$ away from finitely many singular points. The smoothness of the limiting map depends on the estimates that were derived before. Therefore the question is, which of these estimates we still need to control after taking the limit $\varepsilon \rightarrow 0$. In particular, we would like to

1. Keep the number of singularities bounded,
2. Remove the singularities of the solution $(\phi _\infty ,\psi _\infty )$,
3. Control the regularity of the solution $(\phi _\infty ,\psi _\infty )$.

Note that there is no preferred order in which these steps should be performed. However, we cannot expect that the limit $\varepsilon \rightarrow 0$ will exist in general.

### Example 5.1

1. Assume that $M=S^2$ and $N=T^2$. In this case the Euler–Lagrange equations decouple and we have to look for harmonic spinors on $S^2$. It is well-known that these do not exist [2]. Consequently, the limit $\varepsilon \rightarrow 0$ cannot exist in this case and this fact should be reflected by the calculation.
2. If both $M=N=T^2$, the Euler–Lagrange equations also decouple and we have to look for harmonic spinors on $T^2$. The two-dimensional torus has four spin structures and not all of them admit harmonic spinors. Hence, the limit $\varepsilon \rightarrow 0$ cannot be trivial in this case, too.

### Remark 5.2

Both examples show that our approach using the $L^2$-gradient flow of the regularized functional $E_\varepsilon (\phi ,\psi )$ cannot detect the structures associated to the spinor bundle like the spin structure.

### Number of Singularities after $\varepsilon \rightarrow 0$

To study the dependence of the bound on the number of singularities on $\varepsilon$, let us analyze how the bound (3.9) depends on $\varepsilon$. Rearranging (3.9) yields

5.1 $$\begin{aligned} \sum _{i=0}^{j-1}K_i\le C\frac{F(\phi _0,\psi _0)(\varepsilon )}{\delta _1(\varepsilon )}. \end{aligned}$$

It is easy to see that

$$\begin{aligned} \lim _{\varepsilon \rightarrow 0} F(\phi _0,\psi _0)=E(\phi _0)\le C, \end{aligned}$$

but on the other hand the limit

$$\begin{aligned} \lim _{\varepsilon \rightarrow 0}\delta _1(\varepsilon ) \end{aligned}$$

does not exist in general as can easily be seen from the definition of $\delta _1$. Moreover, there is no cancellation of the different $\varepsilon$’s on the right hand side of (5.1).

### Removal of Singularities after $\varepsilon \rightarrow 0$

To remove the singularities of the solution $(\phi _\infty ,\psi _\infty )$ we would like to apply the following (Theorem 4.6 in [17], p. 426):

#### Theorem 5.3

(Removable singularity theorem) For $U\subset M$ let $(\phi ,\psi )$ be a Dirac-harmonic map which is $C^\infty$ on $U{\setminus }\{p\}$ for some $p\in U$. If

$$\begin{aligned} \int _U\big (|d\phi |^2+|\psi |^4\big )dM\le C \end{aligned}$$

then $(\phi ,\psi )$ extends to a $C^\infty$ solution on *U*.

In our case, the $L^2$ norm of $d\phi _\infty$ can be bounded by plugging the spinor $\psi _\infty$ into the inequality for the energy functional $E_\varepsilon (\phi ,\psi )$

$$\begin{aligned} \int _M|d\phi _\infty |^2dM\le E_\varepsilon (\phi _0,\psi _0). \end{aligned}$$

Unfortunately, we cannot bound the $L^4$ norm of $\psi _\infty$ after $\varepsilon \rightarrow 0$.

### Regularity of $(\phi _\infty ,\psi _\infty )$ after $\varepsilon \rightarrow 0$

The regularity of Dirac-harmonic maps has been studied in [38].

#### Definition 5.4

*(Weakly Dirac-harmonic map)* A weak Dirac-harmonic map is a pair $(\phi ,\psi )\in W^{1,2}(M,N)\times W^{1,\frac{4}{3}}{(M,\Sigma M\otimes \phi ^{-1}TN})$, which solves (1.6) and (1.7) in a weak sense.

The relation between weak and smooth Dirac-harmonic maps in dimension two is given by the following ([38], Theorem 1.5, p. 3764)

#### Theorem 5.5

Assume that $M$ is a compact Riemannian spin surface and that the pair $(\phi ,\psi )\in W^{1,2}(M,N)\times W^{1,\frac{4}{3}}(M,\Sigma M\otimes \phi ^{-1}TN)$ is a weak Dirac-harmonic map. Then the pair $(\phi ,\psi )$ is smooth.

Hence, we have to ensure that the estimates necessary for the existence of a weakly Dirac-harmonic map can be carried over to the limit $\varepsilon \rightarrow 0$. Again, the regularity of the map $\phi$ can be assured, but we do not have control over $\psi _\infty$ after $\varepsilon \rightarrow 0$.

## Appendix

The following Lemma combines the pointwise maximum principle with an integral norm. It can be thought of as a simple version of Moser’s parabolic Harnack inequality.

### Lemma A.1

Assume that $(M,h)$ is a compact Riemannian manifold. If a function $u(s,t)\ge 0$ satisfies

$$\begin{aligned} \frac{\partial u}{\partial t}\le \Delta u+Cu, \end{aligned}$$

and if in addition we have the bound

$$\begin{aligned} U(t)=\int _Mu(s,t)dM\le U_0, \end{aligned}$$

then there exists a uniform bound on

$$\begin{aligned} u(s,t)\le e^CKU_0 \end{aligned}$$

with the constant $K$ depending on $M$.

### Proof

A proof can for example be found in [36], p. 284. $\square$
